# Supplementary material for: Computerized physical and cognitive training improves the functional architecture of the brain in adults with Down syndrome: A network science EEG study
Source: Netw Neurosci. 2021 Mar 1;5(1):274–94. doi: 10.1162/netn_a_00177 (PMC7935030; doi:10.1162/netn_a_00177)
Supplement: Supplementary file 1 [file netn-05-274-s001.pdf]

Anagnostopoulou, A., Styliadis, C., Kartsidis, P., Romanopoulou, E., Zilidou, V., Karali, C., Karagianni, M., Klados, M., Paraskevopoulos, E., Bamidis, P. D. (2021). Supporting information for "Computerized physical and cognitive training improves the functional architecture of the brain in adults with Down Syndrome: a network science EEG study." *Network Neuroscience*, *5*(1), 274–294. https://doi.org/10.1162/netn_a_00177

Title: Computerized physical and cognitive training improves the functional architecture of the brain in adults with Down Syndrome: a network science EEG study

Author list:

Alexandra Anagnostopoulou*, Charis Styliadis*, Panagiotis Kartsidis, Evangelia Romanopoulou, Vasiliki Zilidou, Chrysi Karali, Maria Karagianni, Manousos Klados, Evangelos Paraskevopoulos**, Panagiotis D. Bamidis**

* Joined first authorship

** Joined senior authorship

Supplementary Information

The following steps were taken to get a measure of how much of the entropy of the receiver is explained by information flowing from the sender. Values of normalized PTE (dPTE) range between 0 and 1, with weights of node-pairs xy in the interval [0, 0.5) indicating that information flows from x to y, weights in the interval (0.5,1] showcase the opposite direction of information, while weights with a value of 0.5 indicate the equal participation of x and y (Hillebrand et al., 2016). We have averaged the dPTE adjacency matrices per group and then visualized the edges with weights ≤ 0.5 to depict the participation of the receiving node in the information flow i.e., the higher the weight of an edge the more the

receiver participates in the exchange of information. The pre-intervention network seems to have a stronger looping effect in the information flow, as certain receiver nodes participate almost reciprocatively in the exchange of information within the pair. This effect appears to have decreased in the post DS network but an increased amount of receiver nodes seems more engaged in the communication within the network.

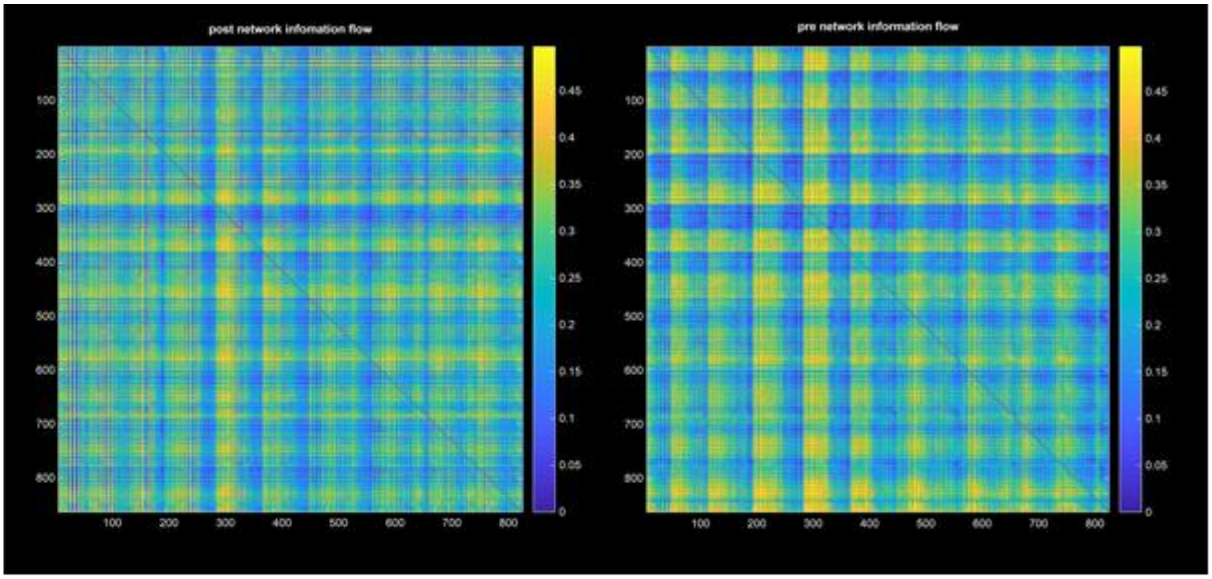

Supplementary Figure 1. Depiction of the engagement of receiver nodes in the exchange of information within the network. Values of normalized PTE (dPTE) range between 0 and 1, with weights of node-pairs xy in the interval [0, 0.5) indicating that information flows from x to y, and weights in the interval (0.5,1] showcase the opposite direction of information, while weights with a value of 0.5 indicate the equal participation of x and y (Hillebrand et al., 2016). The colormap depicts the engagement of nodes receiving information in the communication with their pair ($dPTE \leq 0.5$). The greater the value, the more engaged the receiver node is in the exchange of information.

Supplementary Table 1. Summary of the graph measures used in the analysis

Graph Measures			Definition	Relation to other measures	Use
Global Measures	Measures of integration	Global Efficiency	The average inverse shortest path length. Efficacy of information transference.	Inverse relation to the characteristic path length	Characterization of shifts in integration and cost-efficiency of the network
Global Measures	Measures of integration	Characteristic Path length	The average shortest path length between nodes of the network. Number of edges that need to be traversed for the communication between distant regions of the network.	Inverse relation to global efficiency<br>Related to the small-worldness measure	Characterization of the pre- and post-DS network organization
Global Measures	Measures of segregation	Transitivity	The ratio of closed triplets of nodes to the maximum number of triplets (open and closed).<br>Global clustering organization of the network.	Related to the local clustering coefficient (average clustering coefficient).<br>Related to the small-worldness measure.<br>Does not suffer from individual disproportionate clustering coefficient values	Characterization of the pre- and post-DS network organization, and shifts in the segregation of the network
Local Measures	Measures of segregation	Local Clustering Coefficient	The ratio of a node's connected neighbors to the maximum number of possible connections. Clustering organization in distinct regions that characterizes the robustness of a system.	Related to transitivity	Characterization of shifts in the robustness and fault tolerance of the DS network
Local Measures	Measures of centrality	Node Betweenness Centrality	The fraction of shortest paths that pass through a node.<br>Importance of a node in the information flow	Analogous relation to degree centrality	Characterization of the participation of nodes in the distribution of information

	Network motifs	Node Degree Centrality	Number of links connected to a node. The connectedness of a node to others.	Analogous relation to degree centrality	Computed only for the nodes of the post-DS network, which exhibit a significant increase in connectivity
		Communities	Detection of communities' organization by maximizing modularity. Characterizes the community distribution of a network.		Characterization of the hierarchical organization of the pre- and post- DS networks
		Small-Worldness	The ratio of global clustering coefficient and characteristic path length, normalized by the respective measures of a random network. Characterizes the organization of a network in relation to the threshold of small-worldness ($\sigma>1$ classifies a network as small-world).	Related to the characteristic path length and transitivity	Characterization of the pre- and post-DS network organization.

References

Hillebrand, A., Tewarie, P., van Dellen, E., Yu, M., Carbo, E. W. S., Douw, L., … Stam, C. J. (2016). Direction of information flow in large-scale resting-state networks is frequency-dependent. Proceedings of the National Academy of Sciences of the United States of America, 113(14), 3867–3872. https://doi.org/10.1073/pnas.1515657113
